# Intermittent Hypoxic Therapy Inhibits Allogenic Bone-Graft Resorption by Inhibition of Osteoclastogenesis in a Mouse Model

**DOI:** 10.3390/ijms23010323

**Published:** 2021-12-28

**Authors:** Natasja Leth Bergholt, Ari Demirel, Michael Pedersen, Ming Ding, Tue Wenzel Kragstrup, Thomas Andersen, Bent Winding Deleuran, Casper Bindzus Foldager

**Affiliations:** 1 Orthopaedic Research Laboratory, Aarhus University Hospital, 8200 Aarhus, Denmark; natasja.leth@gmail.com (N.L.B.); ari.demirel@hotmail.com (A.D.); 2 Comparative Medicine Laboratory, Aarhus University, 8200 Aarhus, Denmark; michael@clin.au.dk; 3 Department of Orthopaedic Surgery and Traumatology, Odense University Hospital and University of Southern Denmark, 5000 Odense, Denmark; Ming.Ding@rsyd.dk; 4 Department of Biomedicine, Aarhus University, 8000 Aarhus, Denmark; thomas.andersen@midt.rm.dk (T.W.K.); kragstrup@biomed.au.dk (T.A.); bd@biomed.au.dk (B.W.D.); 5 Department of Rheumatology, Aarhus University Hospital, 8200 Aarhus, Denmark

**Keywords:** intermittent hypoxic therapy, bone, spine

## Abstract

Systemic Intermittent Hypoxic Therapy (IHT) relies on the adaptive response to hypoxic stress. We investigated allogenic bone-graft resorption in the lumbar spine in 48 mice. The mice were exposed to IHT for 1 week before surgery or 1 week after surgery and compared with controls after 1 and 4 weeks. Complete graft resorption was observed in 33–36% of the animals in the control group, but none in the preoperative IHT group. Increased bone-graft volume was demonstrated by micro-computed tomography in the preoperative IHT group after 1 week (*p* = 0.03) while a non-significant difference was observed after 4 weeks (*p* = 0.12). There were no significant differences in the postoperative IHT group. Increased concentration of immune cells was localized in the graft area, and more positive tartrate-resistant acid phosphatase (TRAP) staining was found in controls compared with IHT allogenic bone grafts. Systemic IHT resulted in a significant increase of the major osteoclast inhibitor osteoprotegerin as well as osteogenic and angiogenic regulators Tgfbr3, Fst3l, Wisp1, and Vegfd. Inflammatory cytokines and receptor activator of nuclear factor kappa-B ligand (RANKL) stimulators IL-6, IL-17a, IL-17f, and IL-23r increased after 1 and 4 weeks, and serum RANKL expression remained constant while Ccl3 and Ccl5 decreased. We conclude that the adaptive response to IHT activates numerous pathways leading to inhibition of osteoclastic activity and inhibition of allogenic bone-graft resorption.

## 1. Introduction

Hypoxia is a major universal stimulus able to drive proliferation, differentiation, apoptosis and homeostasis of most cell types through local and systemic oxygen sensing [1]. Local hypoxic cues initiate multiple events responsible for embryogenesis as well as spatial and temporal orchestration of tissue homeostasis [2,3]. Cell survival is dependent on the initial rapid adaptation to hypoxia by hypoxia-inducible factors (HIFs) and mitochondrial reactive oxygen species (ROS) production followed by a prolonged physiological adaptation to hypoxia [4,5]. Chronic hypoxia and long-term or repeated exposure to severe hypoxia, as seen in obstructive sleep apnea, have been linked to diseases such as pulmonary hypertension, while adaptation to the exposure of acute short-term hypoxia has been linked to resistance to tissue injury (e.g., in myocardial infarction) [6,7]. The adaptive response to short-term hypoxia is also being used in endurance athletes to enhance athletic performance and recovery [8]. In this study, we define systemic intermittent hypoxic therapy (IHT) as short periods of exposure to lower-than-normal levels of oxygen in the inspired air interrupted by recovery periods.

Bone-graft resorption is a major issue in spinal fusion surgeries leading to treatment failures and a potential need for reoperation. Bone homeostasis and regeneration are driven by a cross-talk between osteoblasts and osteoclasts. A dysregulated cross-talk leads to diseases such as osteoporosis and osteopetrosis. Interventional therapies addressing the interplay between osteoblasts and osteoclasts are used in regenerative orthopaedics, a dynamic process that balances formation of new bone, cell survival, and integration of bone grafts, bone-graft substitutes, and neoangiogenesis within these areas. Osteoclasts are multinucleated terminally differentiated myeloid cells adapted to remove mineralized bone matrix, and osteoclast precursors express receptor activator of nuclear factor-κB (RANK), the receptor for the RANK ligand (RANKL). Osteoprotegerin (OPG) is a decoy receptor of RANKL and is mainly synthesized by osteoblasts and mature B-cells in the bone marrow. RANKL/OPG expression ratio determines the osteoclast differentiation and function, and this ratio is considered a major determinant of bone turnover.

Recently, it has become evident that immunogenic cells play important roles in regulation of bone homeostasis. The complexity of this system is illustrated by the dual role of interleukin-17 (IL-17) in immune-mediated inflammatory arthritis. Thus, IL-17A and Th17 cells are linked to bone resorption in the peripheral joints of patients with inflammatory arthritis [9]. However, IL-17A has also been shown to induce BMP-2, osteoblastogenesis and mineralization, and the combination of BMP-2 and IL-17A is a potent inducer of osteogenesis [10,11,12]. The clinical efficacy of anti-IL-17A antibodies in ankylosing spondylitis also indicated in the axial joints that IL-17A is predominantly related to new bone formation [13].

The aim of this study was to evaluate the effect of IHT on allogenic bone-graft survival and new bone formation in mice and to investigate the relationship between the humoral response and bone homeostasis. We hypothesized that the adaptive systemic response to exposure to 1 week of IHT pre- or postoperatively would be a cascade of anabolic and immunomodulatory events resulting in increased bone and allogenic bone-graft survival.

## 2. Results

### 2.1. Less Bone Resorption and Reduced Osteoclast Activity

**Figure 1:**
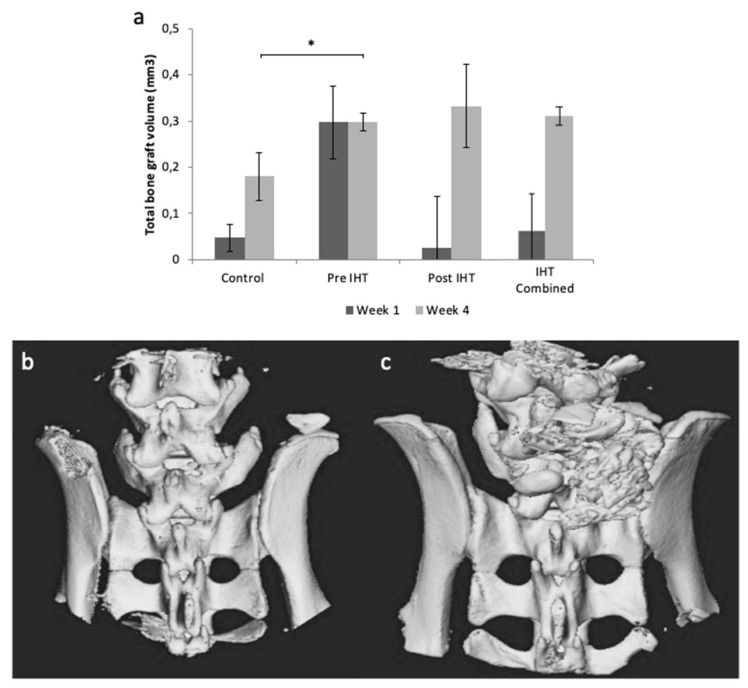
Intermittent Hypoxic Therapy (IHT) reduced bone resorption in vivo. Male C57BL/6 mice 6–10 weeks old receiving implantation of 10 mg allogenic bone graft posterior to the lumbar spine. Total bone-graft volume showed to be holding a prominent position within IHT groups. (**a**) Total bone-graft volume for control, pre IHT, post IHT, and IHT groups combined at 1 and 4 weeks. Three-dimensional reconstructions from μCT visualizes bone resorption: (**b**) representative μCT image from control 1 week postoperative, and (**c**) representative μCT image from pre IHT 1 week postoperative. Controls: *n* = 11, biological independent samples. IHTs: *n* = 6, biological independent samples. (*) represents statistical significance (*p* < 0.05).

Microcomputed tomography (μCT) was performed to investigate total bone-graft volume. One animal in the control group was excluded due to infection. At 1-week postoperative, complete bone-graft resorption was observed in 0/6 (0%) mice in the pre IHT group, 1/6 (17%) mice in the post IHT group, and 4/11 (36%) mice in the control group. At 4 weeks postoperative, complete graft resorption was observed in none (0%) of the IHT groups and 4/12 (33%) in the control group. Representative 3-dimensional reconstructions of μCT images are presented in Figure 1b,c. Total bone-graft volume was higher in both IHT groups compared with controls (Figure 1a), and this finding was significant for the preoperative IHT group after 4 weeks (*p* = 0.003) (Figure 1a).

**Figure 2:**
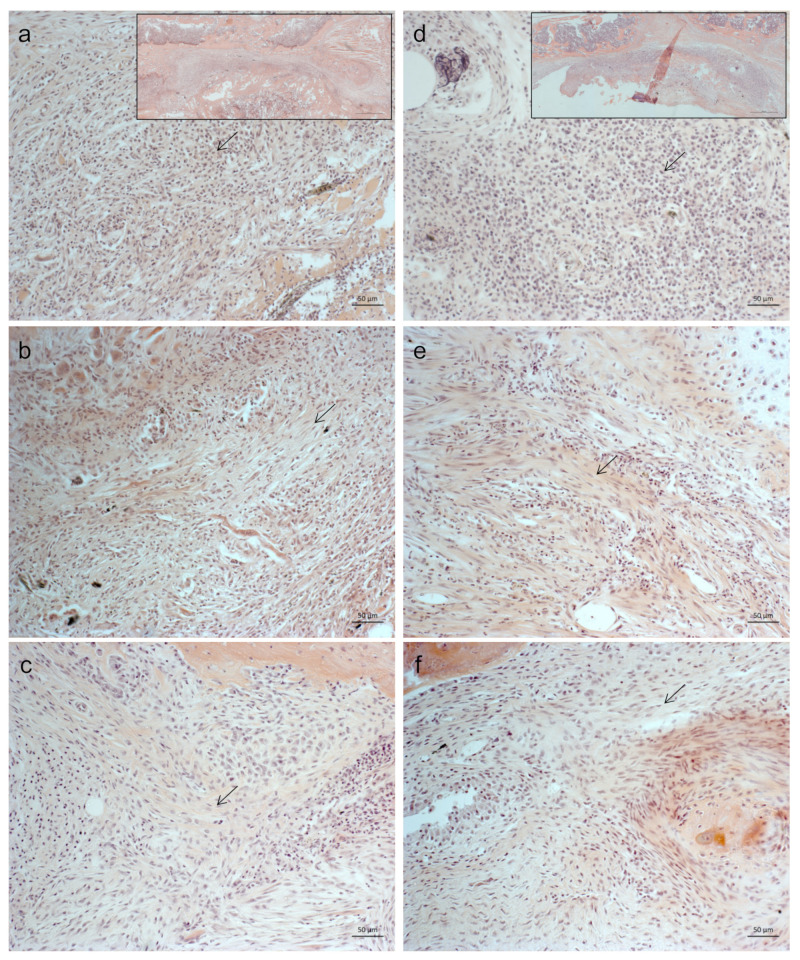
Intermittent Hypoxic Therapy (IHT) mitigates immune-cell infiltration of the allogenic bone-graft tissue of mice. Morphological representative images of hematoxylin- and eosin (HE)-stained histological sections of the allogenic bone-graft area at 1 week (**a**–**c**) and 4 weeks (**d**–**f**) for controls (**a**,**d**), pre IHT (**b**,**e**), and post IHT (**c**,**f**). Note that Arrows indicate the superiority of cells within the representative image. Scale bar 50 μm. The marked squares in the top row of (**a**,**d**) represent HE stained images of 6- to 10-week old male C57BL/6 mice receiving implantation of 10 mg allogenic bone graft posterior to the lumbar spine (control) after 1 and 4 weeks. Scale bar 500 μm.

Hematoxylin and eosin (HE) staining was used for morphological evaluation of the allogenic tissue. The reactive graft demonstrated aggregates of distinctive eosinophilic nuclei, immune cells, contrasts spindle-like or elongated shaped nuclei, predominantly found in connective tissue. We observed darkly stained nuclei aggregates of leukocytes in all treatments and stages of healing. However, the tendency was a reduced number of immune cells within the reactive graft tissue when treated with IHT (Figure 2).

**Figure 3:**
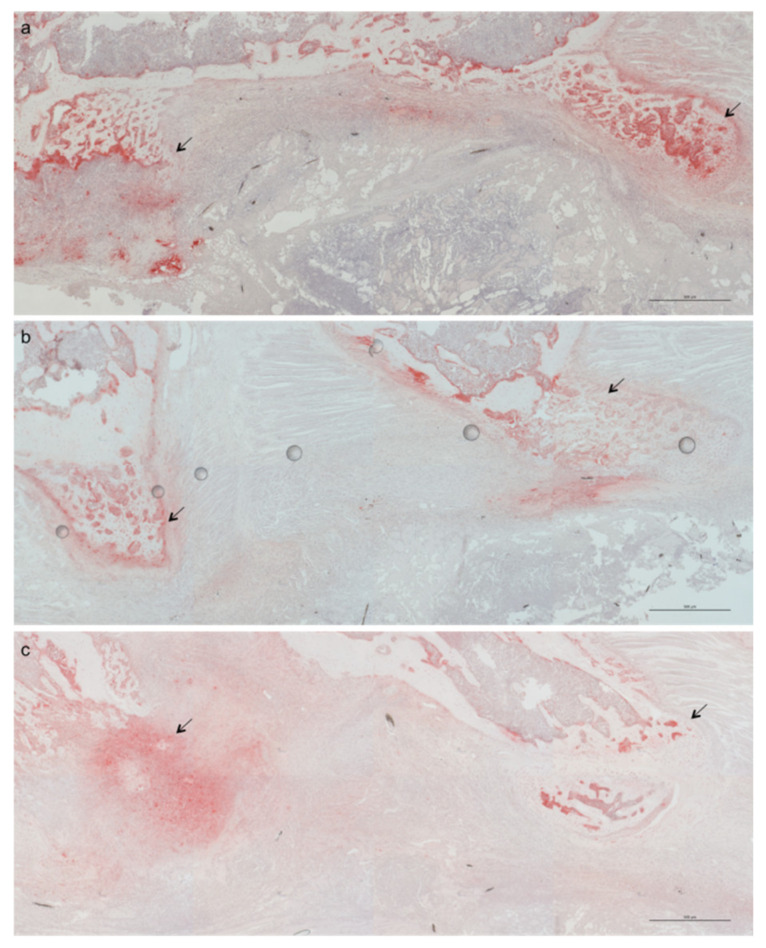
Intermittent Hypoxic Therapy (IHT) reduced osteoclastogenesis. Tartrate-resistant acid phosphatase (TRAP)-stained histological sections of allogenic bone-graft areas after 1 week showed strong TRAP staining of osteoclasts in control mice. Groups included control (**a**), pre IHT (**b**), and post IHT (**c**). Scale bar 500 μm. Arrows indicate areas of positive TRAP staining.

The tissue morphology showed fibroblastic features in groups treated with IHT compared with controls. However, it was impossible to discriminate between the IHT groups. Because activation of osteoimmunological cascades mediated by IHT might disturb native bone homeostasis, we stained the presence of osteoclast activity using tartrate-resistant acid phosphatase (TRAP). One week postoperative, TRAP was abundantly expressed in the bone areas of control compared with IHT groups, suggesting an increased osteoclastogenesis in controls and relatively reduced osteoclast activity in the IHT groups (Figure 3).

**Figure 4:**
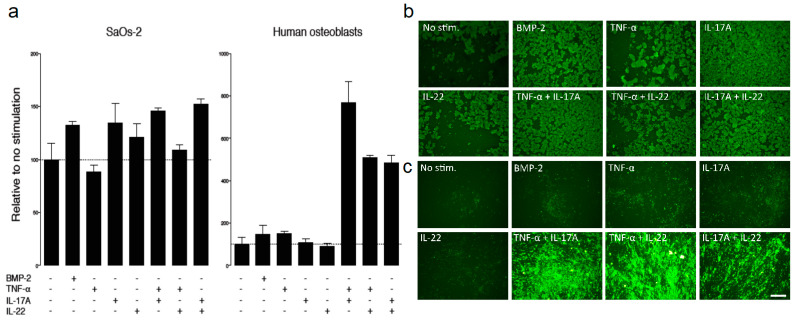
Bone mineralization of osteogenic cells depends on protein interplay. (**a**) SaOs-2 and human osteoblasts were cultured with different protein families such as BMP-2, TNF-α, IL-17A, and Il-22. Vertical axes represent relative to no stimulation. Horizontal axes represent different protein stimulation of SaOs-2 and human osteoblasts. The interplay of proteins displays the highest expression. (**b**,**c**) Visualization of bone mineralization under cytokine stimulation. (**b**) SaOs-2 cells and (**c**) human osteoblasts. All values are relative to the control cell with no stimulation (No stim). Scale bar 250 μm.

Osteoblasts are responsible for bone mineralization. We investigated the influence of several proteins that belong to cytokine receptor superfamilies, including IL, TGF, and TNF, responsible for bone mineralization. BMP2 is a potent inducer of bone formation [14]. However, the immunological interplay between TNF-α and IL-17A or IL-22 was found to induce stronger bone mineralization compared with BMP2 alone (Figure 4a). Upon stimulation with TNF-α and IL-17A, human osteoblasts had the strongest intensity compared with the SaOs-2 cell line, demonstrating the importance of local protein interplay but also an osteoimmunological response of human osteoblasts under atmospheric oxygenic conditions (Figure 4a–c).

### 2.2. A Diverse Exploratory Blood Signature of IHT

**Figure 5:**
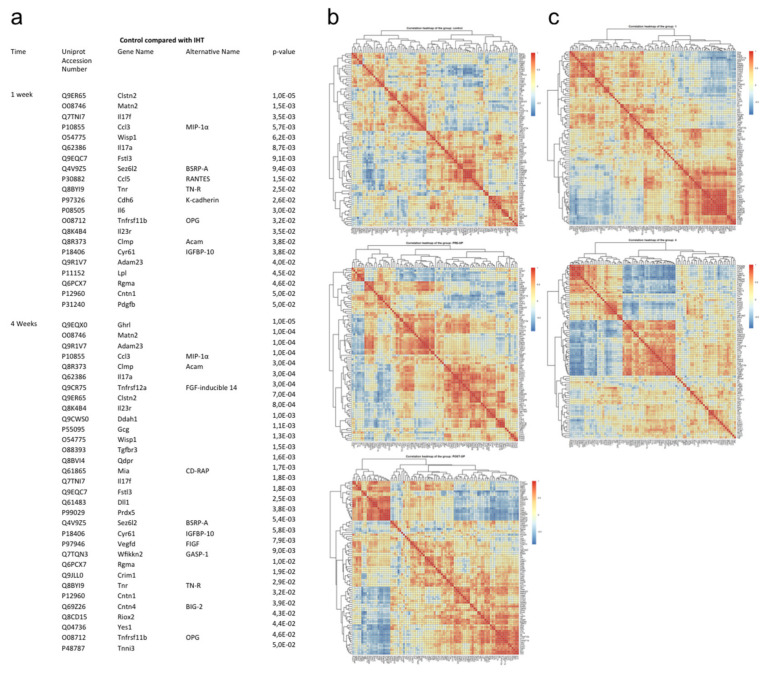
Systemic explorative proteins of interest. (**a**) Proteins and their gene abbreviations that showed significant difference using one-way ANOVA or the Kruskal–Wallis rank test. Data are shown as unadjusted NPX data, control versus Intermittent Hypoxic Therapy (IHT). Abbreviations: Calsyntenin 2, Clstn2; Matrilin-2, Mats2; Interleukin-17F, Il17f; C-C motif chemokine 3, Ccl3; WNT1-inducible-signaling pathway protein 1, Wisp1; Interleukin-17A, Il17a; Follistatin-related protein 3, Fstl3; Seizure 6-like protein 2, Sez6l2; C-C motif chemokine 5, Ccl5; Tenascin-R, Tnr; Cadherin-6, Cdh6; Interleukin-6, Il6; Tumor necrosis factor receptor superfamily member 11B, Tnfrsf11b; Interleukin-23 receptor, Il23r; CXADR-like membrane protein, Clmp; Protein CYR61, Cyr61; Disintegrin and metalloproteinase domain containing protein 23, Adam23; Lipoprotein lipase, Lpl; Repulsive guidance molecule A, Rgma; Contactin-1, Cntn1; Platelet-derived growth factor subunit B, Pdgfb; Appetite-regulating hormone, Ghrl; Tumor necrosis factor receptor superfamily member 12 A, Tnfrsf12a; N(g),N(G)-dimethylarginine dimethylaminohydrolase 1, Ddah1; Glucagon, Gcg; Transforming growth factor beta receptor type 3, Tgfbr3; Dihydropteridine reductase, Qdpr; Melanoma-derived growth regulatory protein, Mia; Delta-like protein 1, DII1; Peroxiredoxin-5, mitochondrial, Prdx5; Vascular endothelial growth factor D, Vegfd; WAP Kazal immunoglobulin Kunitz and NTR domin-containing protein 2, Wfikkn2; Cysteine-rich motor neuron 1 protein, Crim1; Contactin-4, Cntn4; Ribosomal oxygenase 2, Riox2; Tyrosine-protein kinase Yes, Yes1; Troponin I, cardiac muscle, Tnni3. (**b**,**c**) Clustered heatmaps and the correlation between protein expression within groups and time were determined using the Pearson’s correlation analysis across all NPX values. *n* = 12, biological independent samples. (**b**) Association of protein expression within groups. Column order: control, pre IHT and post IHT. (**c**) Association of protein expression within time. Column order: 1 week and 4 weeks. The Pearson’s correlation analyses were derived in R version 3.6.1.

Using the Olink exploratory platform, we assessed an unbiased panel of 92 murine blood proteins in serum of mice operated on in the lumbar spine and treated with IHT preoperatively or postoperatively compared with control over time [15]. Among the 92 proteins, 21 proteins were significantly regulated in IHT compared with the control group after 1 week (Figure 5a). After 4 weeks, 32 proteins were significantly regulated (Figure 5a). The majority of these proteins were located to the extracellular region. The significantly regulated proteins were exposed to STRING (version 11.0) interaction mapping. Regulation of osteoclast differentiation, chemotaxia, and inflammatory response were among the biological processes and GO terms that were regulated in response to IHT after 1 week (Supplementary Materials Figure S1). Proteins indicative of stimulation of osteoclast differentiation were IL-17A, CCL3, and CCL5 while proteins involved in osteoclast inhibition were IL-6, Fstl3, and Tnfrs11b (OPG). Four weeks postoperative, angiogenesis was among the biological mechanisms that was particularly regulated based on the protein profile (Supplementary Materials Figure S2). Heat maps represent Pearson’s correlation between systemic protein expression based on NPX values of identified protein within groups (control, pre IHT, and post IHT groups) and over time (Figure 5b,c).

The unbiased panel of 92 proteins clustered into communities that were closely correlated, positively or negatively. Pre IHT and post IHT provided detailed insight in modules with high positive protein correlation concomitant with negative protein correlation (red, positive correlation; blue, negative correlation). In summary, this finding showed interconnection of proteins within similar or reverse expression patterns and potential co-dependency. Several proteins were affected by IHT, and time also shifted the systemic protein expression pattern of correlation. Furthermore, differences within the IHT groups were also observed and investigated for their osteogenic and angiogenic responses.

**Figure 6:**
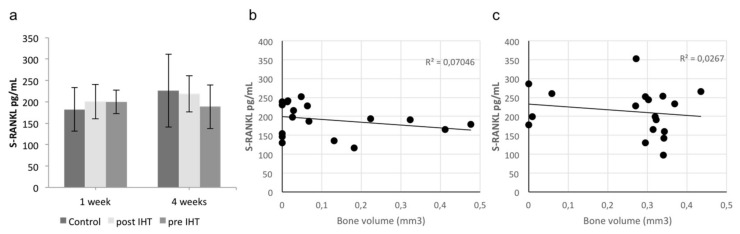
Osteoclast stimulating factor in serum. (**a**) RANKL expression in serum after 1 and 4 weeks. Vertical axis represents p-RANKL concentrations (pg/mL). Horizontal axis represents time visualized by different treatment groups. Average level was 200 pg/mL (range 97–350 pg/mL). No differences were observed between the groups. (**b**,**c**) RANKL and bone-volume correlation after 1 week and 4 weeks. Vertical axes represent S-RANKL concentrations (pg/mL). Horizontal axes represent bone volume (mm^3^). Correlation coefficients were investigated for the clustered groups at each time point. No correlation between bone-graft volume and RANKL concentration in serum was found. Control, *n* = 12 biological independent samples. IHTs, *n* = 6 biological independent samples.

Bone formation and bone-graft survival are dependent on osteoblastic–osteoclastic interplay and angiogenesis. RANKL is the primary stimulator of osteoclast activity. We found no differences in the expression of systemic RANKL in response to IHT (Figure 6).

Osteoprotegerin (OPG; Tnfrs11b), a decoy receptor for RANKL that promotes inhibition of osteoclastogenesis, was significantly induced after 1 week of IHT (*p* = 0.03) and at 4 weeks (*p* = 0.04). The expression was significantly higher at 1 week postoperative compared with 4 weeks. We found no differences between the two hypoxic treatments (Figure 6).

**Figure 7:**
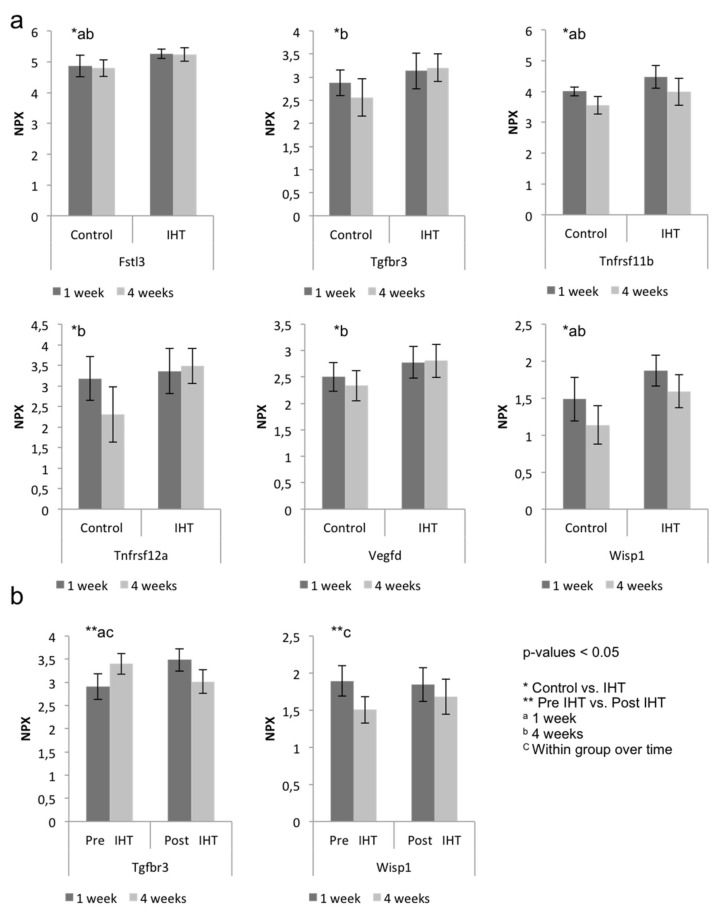
Systemic expression of osteo- and angiogenic proteins. Log2-transformed arbitrary units normalized protein expression (NPX) of selected proteins after 1 and 4 weeks. (**a**) Control compared with Intermittent Hypoxic Therapy (IHT). Osteogenic and angiogenic factors were regulated using IHT, and differences were observed over time. Vertical axes represent NPX values. Horizontal axes represent treatment groups visualized over time. *n* = 12, biological independent samples. (**b**) pre IHT compared with post IHT. In particular, Tgfbr3 was upregulated at post IHT and within the same IHT but over time. Vertical axes represent NPX values. Horizontal axes represent treatment groups visualized over time. *n* = 6, biological independent samples. Statistical analysis was performed using one-way ANOVA or the Kruskal–Wallis rank test. Data are presented as unadjusted NPX data, * control compared with IHT or ** pre IHT compared with post IHT. ^a^ 1 week and ^b^ 4 weeks. *p*-values < 0.05 were considered statistically significant.

Betaglycan, a transforming growth factor beta receptor 3 (Tgfbr3), is an accessory receptor that modulates the ligand-binding specificity. It is capable of binding all three TGF-β types and BMP2 and is found differentially expressed in subjects with normal versus abnormal fracture healing and to regulate angiogenesis and osteoblast development during palatogenesis [16,17]. We found that Tgfbr3 was significantly increased by IHT after 4 weeks (*p* = 0.002) (Figure 7a), and we found a difference between the 2 IHT groups 1 week postoperative compared with the post IHT group having significantly higher expression of Tgfbr3 (*p* = 0.003) (Figure 7b).

Follistatin-related protein 3 (Fstl3), a mediator of exercise-driven bone formation and fracture resistance, was significantly regulated after 1 week (*p* = 0.009) and 4 weeks (*p* = 0.002) [18]. According to the GO-term database, Fstl3 is involved in regulation of osteoclast differentiation (Supplementary Materials Figure S1).

The WNT1-inducible signaling-pathway protein 1 (Wisp1) is a member of the connective tissue growth factor (CTGF) family. A wide range of cell types synthesize Wisp1, and its role is highly tissue-specific. In bone, Wisp1 is synthesized by osteoblasts and its precursors, and plays a role in regulating bone mineral content, cortical and trabecular bone thickness, and bone strength during aging by modulating osteoblast and osteoclast function, presumably via regulation of osteogenic differentiation of bone-marrow stromal cells [19]. We found that Wisp1 was significantly increased by IHT after 1 week (*p* = 0.006) and 4 weeks (*p* = 0.001). Furthermore, Wisp1 was significantly higher expressed in pre IHT after 1 week compared with 4 weeks (*p* = 0.03). Vascular endothelial growth factor d (VEGFD) is a ligand of Vegfr3 under the control of Vegf expression. Studies have shown that VEGFD is critical in osteoblast maturation and is also suggested to be a downstream effector of VEGF in osteogenesis [20]. We found significant induction by IHT after 4 weeks (*p* = 0.008). We observed a tendency of stronger angiogenic expression over time within IHT, such as Vegfd, Wisp and Tgfbr3, and a higher inhibitory presence of osteoclastogenesis modulators, including Fstl3 and Tnfrsf11b in the IHT groups compared with controls, suggesting a systemic activation by IHT.

### 2.3. IHT Promotes an Osteoimmunological Response

**Figure 8:**
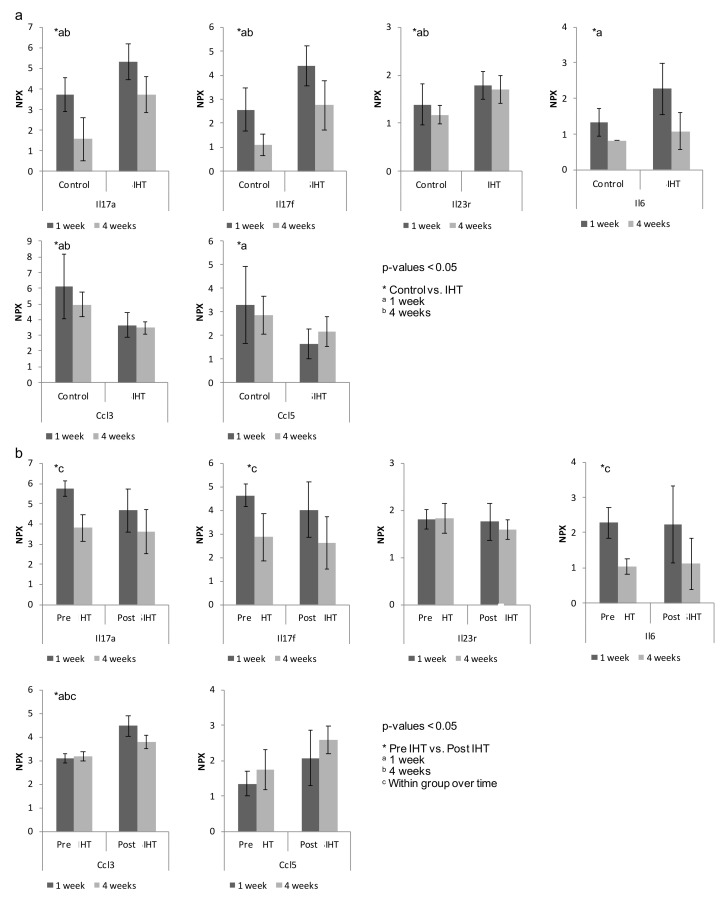
Intermittent Hypoxic Therapy (IHT) induces systemic osteoimmunological expression compared with control. Log2-transformed arbitrary units normalized protein expression (NPX) of selected proteins after 1 and 4 weeks. The cytokines (ILs) had the highest response within IHT after 1 week, while the chemotactic cytokines (Ccls) were downregulated in IHT at both time points. Vertical axes represent NPX values. Horizontal axes represent treatment groups visualized over time. *n* = 12, biological independent samples. Horizontal axes represent treatment groups visualized over time. *n* = 6, biological independent samples. Statistical analysis was performed using one-way ANOVA or the Kruskal–Wallis rank test. Data are presented as unadjusted NPX data, (**a**) * control compared with IHT or (**b**) * pre IHT compared with post IHT. ^a^ 1 week, ^b^ 4 weeks, and ^c^ within IHT over time. *p*-values < 0.05 were considered statistically significant.

Surgery induces an inflammatory response, and we performed a proteomic analysis to elucidate the plethora effects induced by IHT. We investigated the inflammatory component induced by IHT at 1 and 4 weeks postoperative relative to controls. Interleukins are known to play a critical role in the immune response. Significant changes were induced in the majority of interleukins synthesized and secreted by CD4^+^ T cells, monocytes, and macrophages. Of the cytokines that showed the greatest changes by IHT, many were related to the TH17-subset, including IL-6, IL-17A, IL-17F and IL-23R (Figure 8). We found that IL-6, IL-17A and IL-17F were increased by IHT at both 1 and 4 weeks compared with controls. The immunological response of the interleukins IL-17A, IL-17F and IL-23R was significantly higher at 1 week postoperative compared with 4 weeks for all groups, with a tendency towards the highest expression in the IHT groups (Figure 8). CCL3 macrophage inflammatory protein (MIP-1) has previously been found to stimulate osteoclast differentiation in bone remodeling [21]. We found an upregulated level of CCL3 in control compared with IHT after 4 weeks. Relative to the interleukins, an inverse tendency was observed for the chemotaxis proteins CCL3 and CCL5. These chemokines were reported to be pro-inflammatory and to stimulate the recruitment of osteoclast precursors for osteoclastogenesis, and CCL5 has been shown to influence new bone formation by chemotaxis of mesenchymal stem cells [22]. This finding indicates a reduced chemotaxis of osteoclast cell recruitment following IHT exposure. We observed a higher NPX expression of interleukins in serum from IHT groups after 1 week, but a higher number of immune cells localized at the bone-graft areas in the controls, suggesting a systemic response of IHT that induces immunological protein modulation that potentially favors systemic healing compared with local healing of the graft area.

## 3. Discussion

Systemic adaptation to intermittent hypoxic stress is known as one of the main benefits of physical exercise. This adaptation occurs within minutes and is characterized by ubiquitous events of catabolic, anabolic, pro- and anti-inflammatory-, and regulatory cellular events. We found a significant long-lasting humoral adaptation in response to IHT, resulting in increased allogenic bone-graft survival in mice. Bone-graft resorption is a major issue in spinal fusion surgeries, leading to treatment failures and potential needs for secondary surgery. Complete bone-graft resorption in instrumented posterolateral spinal fusion is found in one-third of all patients, and nonunion rates in uninstrumented spinal fusion are often reported for one-third to half of all patients [23,24]. Activation of osteoblasts by parathyroid hormone has not resulted in improved bone-graft survival and fusion rates [25]. Uncoupled bone remodeling is due to impaired balance between osteoblasts and osteoclasts in bone. Osteoclast differentiation is under the control of cytokines; among those are RANKL, OPG and macrophage-colony stimulating factors produced by osteoprogenitor cells and macrophages in response to regulatory molecules. Excessive inhibition of RANKL-initiated osteoclastic activity through topical administration of bisphosphonates has resulted in prevention of allograft bone integration and poor implant fixation in a canine model [26]. Hence, a unilateral approach targeting osteogenesis and allogenic bone-graft remodeling may be insufficient. We found less bone-graft resorption with IHT. Importantly, the biological mechanism associated with systemic intermittent hypoxic therapy involves 37 proteins, including 12 proteins that are involved in the interplay of bone formation, angiogenesis, and the immune system. Many of these proteins had a significantly different temporal expression compared with controls, suggesting a long-term response of IHT that favors osteoimmunological regulation. We found significant differences in anabolic and immune regulators from the major protein superfamilies (Il, Tgf, Vegf and TNF), which are strongly associated with the osteoimmunological response. Transforming growth factor-β1 (Tgfb1) is non-covalently bound to latency-associated protein (LAP) in bone matrix. The Tgfb1/LAP complex is bound to the latent Tgfb1-binding protein and secreted from cells bound to collagen and other matrix molecules. The active form is released during bone resorption and stimulates mesenchymal osteoprogenitor cells for osteoblastic differentiation, and thus acts as the primary coupling factor between osteoclast activity and osteoblastic differentiation [27,28]. Active Tgfb1 release is also seen from platelets upon activation [29]. We found no significant differences in Tgfb1 expression following IHT exposure. Bone-marrow stromal cells, osteoprogenitor cells and mature osteoblasts express the two types of type III, or accessory, receptors [30,31]. Tgfb1 binding to the Tgfb1receptor activates Smad2 and Smad3 as well as the common Smad4, while Smad-independent pathways also occur [32,33]. The type-III receptor, Tgfbr3, which was significantly upregulated by IHT, exists in a membrane-bound and a soluble form. The latter, which is included in the present study, is likely released by cells upon proteolysis and inhibits TGF-β signaling by sequestering [30]. In addition to its role as an osteoblastic–-osteoclastic coupling factor, Tgfb1 also plays a critical role in the regulation of CD4+ T cells [34]. Naïve CD4+ T cells can also differentiate into four different subgroups of helper cells. In addition to the classic Th1 and Th2 cells, CD4+ T cells are differentiated into Th17 cells upon stimulation by TGFβ and IL-6 or IL-21, and these cells are characterized by their secretion of IL-17A and IL-17F [35,36]. IL-17 secretion from Th17 cells stimulates epithelial, endothelial, and fibroblasts to secrete pro-inflammatory factors such as IL-6, IL-8, GM-CSF, CXCL1 and CCL20. The increased expressions of IL-17A and IL-17F by IHT in the present study indicate initiation of an inflammatory immunologic cascade as part of the adaptive response to the hypoxic therapy. IL-17A and IL-17F share the highest-degree sequence homology among the IL-17 subtypes and are originally regarded as pro-inflammatory cytokines belonging to the TH17 subset. The differentiation towards TH17 is initiated by stimulation by IL-6 or IL-1, in combination with TGFb, and their growths are sustained by IL-23 [37,38]. IL-23 is required for Th17 cells’ maintenance and has also been suggested to inhibit osteoclastogenesis through T-cell stimulation and to improve trabecular bone mass in control mice compared with IL-23 lacking the p19-subunit [39,40,41]. IL-6 has, however, also been found to affect non-immunogenic cells and to stimulate RANKL responsible for differentiation and activation of osteoclasts. Thus, IL-6 is not essential for bone resorption [40].

We found a decreased expression of CCL3 and CCL5 by IHT. CCL3 and CCL5 are chemoattractant molecules that affect osteoclast precursors and potentiate the activity of osteoclasts by RANK and IL-6 [42,43]. However, they inhibit osteoblast activity through regulation of transcription factors Runx2 and Osterix [44]. We argue that the reduced serum level of the attraction content for precursors of osteoclast inheritance is reflected in the reduced positive TRAP detection and recruitment of leukocytes into the local tissue environment of the allogenic bone sites. Nonetheless, the proximity of osteoimmunologic interplay between osteoblasts and osteoclasts is not fully understood. Collectively, these findings suggest a highly complex interplay between these cytokines and their expression co-dependency, leading to challenges in understanding their functional role without considering potential degrees of omnipotence. We have previously shown the adaptive response to short-term IHT is dependent on age. It is plausible that several additional factors (gender, BMI, muscle mass, metabolic cues, etc.) are important determinants of the individual adaptive response to IHT. Similarly, one could hypothesize that individuals are more or less inflammatory-prone at the initiation of treatments, leading to diverse experience and outcomes following IHT exposure. Thus, clinical application of IHT may need individualized protocols. We observed low total bone-graft volume in the post IHT group relative to the pre IHT group at 1 week postoperative, which may be due to the timing of the IHT intervention. The data distribution is, however, high in this group, and causality remains speculative. At 4 weeks postoperative, both IHT groups presented high total bone-graft volumes, which is a result of reduced resorption and potentially increased new bone formation as several factors involved in new bone formation were upregulated as discussed below. In the post IHT group, the outcome at 4 weeks was a result of 4 mice with high bone volume and 2 mice with almost complete bone resorption reflected in the high standard deviation. Naturally, the ability to recover from almost-complete bone-graft resorption at early time points is biologically troublesome, and the avoidance of early resorption is an important experimental and clinical objective in spinal fusion research. We observed nonsignificant differences in bone-graft resorption and total volume between the IHT groups. Timing of the hypoxic stimulation and subsequent systemic and local molecular responses may thus be a contributing factor in the overall outcome.

The adverse effect of excessive exposure to chronic hypoxia or repeated severe intermittent hypoxia has been described in people living at high altitude and in patients suffering from obstructive sleep apnea. Limited periods of intermittent exposure of moderate systemic hypoxia, however, have not demonstrated side effects clinically or experimentally. Positive systemic effects, such as in blood pressure and bone mineral density, have been found, but further investigations are needed to clarify causality [45]. We investigated systemic concentrations of proteins using an unbiased predefined panel. Hence, the study is limited by the array of pre-selected proteins and selected additions investigated by ELISA. The magnitude of a range of anti-inflammatory cytokines produced in the adaptive response remains unknown. Furthermore, while systemic effects are expected to participate in the regulation of bone formation and bone-graft survival, the concentrations in the local environment are responsible for these effects. An advantage of this study is the predefined protein profiling, which only strengthened the exploratory effect of IHT and gained interest in the involved protein and their pathways.

Overall, our study revealed a long-lasting humoral adaptation in response to IHT, resulting in an increased allogenic bone-graft survival in a mouse model. Our findings suggest that IHT might have a substantial effect on the osteoimmunological responses of bone homeostasis. However, IHT also fostered expression of strong bone-formation markers such as OPG, Wisp1, and Fstl3. These markers target bone formation from different areas by competitive ligand interaction, and exercise driven bone formation and microenvironment interaction on osteoblasts. Clinical translation of these findings may help to reduce allogenic bone-graft resorption and improve bone-graft survival in orthopaedic surgeries.

## 4. Materials and Methods

### 4.1. Study Procedures

A pragmatic study design was applied in order to first investigate the in vivo effects of IHT on allogenic bone graft and subsequently apply in vitro investigations to explain the potential findings. We implanted 10 mg of allogenic bone graft posterior to the lumbar spine in 48 6–10 week old male C57BL/6 mice (Janvier Labs) with. The mice were divided into three groups. The 2 intervention groups received 1 week of IHT sessions either 1 week preoperative (pre IHT) or 1 week postoperative (post IHT). One group served as the control group. The groups were followed for 1 week (*n* = 6 in IHT groups and *n* = 12 in control group) and 4 weeks postoperative (*n* = 6 in IHT groups and *n* = 12 in control group). Prior to the surgical intervention, allogenic bone graft was prepared from 6 mice of the same strain as the experimental groups. These mice were euthanized by cervical dislocation, and the femurs were stripped of soft tissue bilaterally. The cartilage-covered parts were discarded. Small pieces of bone graft were cut with microsurgical scissors and transferred into a small cup where graft material was homogenized in size by being crushed with a blunt probe of stainless steel. Individual vials of bone graft containing 10 mg/vial were stored at −80 °C until use.

All mice underwent surgery in general anaesthesia induced and maintained by sevoflurane (Sevorane, AbbVie, Lake Bluff, IL, USA). They received a preoperative intramuscular injection of 0.3 mg/mL Temgesic (Temesic, Indivior PLC, Chesterfield, VA, USA) and the eyes were protected with Lubrithal (Dechra, Uldum, Denmark). The mice were placed in a prone position, and the surgical site was shaved with an electric razor and prepped twice with 96% ethanol. Using a surgical colposcope (Carl Zeiss, Jena, Germany) the lumbar segments L5-L6 were identified by iliac crests, and a 2 cm longitudinal midline incision was made along the lumbar spine. The skin, subcutaneous adipose tissue, and fascia were incised using an x-blade scalpel. A microdissection surgical scissors was used to loosen the muscles from the posterior of the lumbar vertebra. Following identification of the L5-L6 level, gentle decortication was performed bilaterally with a Microlance 3 (Becton, Franklin Lakes, NJ, USA). The thawed bone graft was placed bilaterally on each side of the spinous processes at the L5-6. Finally, fascia, subcutaneous tissue, and skin were sutured with 6-0 microlance (Medtronic, Dublin, Ireland). Each mouse was treated for with 0.3 mg/mL Temgesic for 24 h postoperatively. The mice were allowed to move freely immediately following surgery. They were housed in individual cages in 12:12 light–-dark cycles. The animals were sacrificed at 1 and 4 weeks postoperative for investigations. Euthanasia was performed by exsanguination through the left ventricle of the heart [46].

### 4.2. Intermittent Hypoxic Therapy (IHT)

A normobaric hypoxic chamber was modified to replace atmospheric oxygen by reducing oxygen through a nitrogen membrane unit. The system was set to 52% reduction of atmospheric oxygen corresponding to oxygen concentrations of 10%. The mouse cages were placed in the precalibrated hypoxic chamber for 30 min with 12-h intervals for 1 week either pre- or postoperatively. The opening and closing of the small doors of the hypoxic chamber did not affect the overall oxygen levels in the chamber. This was ensured by continuous measuring of the oxygen level inside the chamber.

### 4.3. Tissue Preparation

Following euthanasia, L4-L6 was dissected, and tissue samples were fixed and dehydrated in a graded series of ethanol (70–96%) followed by clearing in isopropanol and xylene. Finally, the samples were embedded in methoxymethylacrylate (MMA) and prepared for μCT scanning and histology.

### 4.4. Microcomputed Tomography (μCT)

For all mice, the lumbar spines (L4-L6) were harvested and analyzed using ultra-high-resolution μCT system (μCT model 50; Scanco Medical, Wangen-Brüttisellen, Switzerland). Samples were scanned in 70% ethanol with a voltage of 90 kV, current of 155 μA and high integration time of 1500 ms to acquire optimal images. Each 3-D image dataset consisted of approximately 1500 μCT slices, and the scanned images had 3-D reconstruction cubic voxel sizes of 6 × 6 × 6 μm^3^ (5880 × 5880 × 1491 voxels) with 32-bit-gray-levels. For field of view, the scan diameter was 5880 pixels×6 μm/pixel = 35 mm, and the volume was 35 × 35 × 6 mm^3^. The post-operative bone graft and bone fusion identified with assumption-free 3-D methods using Scanco software. After contouring, bone volume (BV), total volume (TV) and bone volume/total volume (BV/TV) were assessed for each sample from 3D μCT image slices. Bone volume fraction (BV/TV, %) was calculated as bone volume per total volume of sample [47].

### 4.5. Histology

MMA-embedded samples were cut in sagittal orientation in 7-μm thick slices on a hard-tissue microtome and mounted on microscopic slides. The sections were deplastified and rehydrated in a graded series of ethanol and rinsed in water. Tissue morphology was evaluated on hematoxylin- and eosin (HE)-stained sections. Osteoclasts were stained using the histochemical marker of osteoclasts tartrate-resistant acid phosphate (TRAP). Briefly, sections were incubated with TRAP basic solution of sodium acetate anhydrous and L-Tartaric acid dissolved distilled water pH 5, followed with naphthol AS-BI-phosphate dissolved in ethylene glycol monoethyl ether incubation at room temperature for 45 min. Five minutes before ending, sodium nitrite solution and pararosaniline chloride were added. Tissue morphology and histochemistry were evaluated by microscope (Zeiss Axio Imager M2 and Zeiss Axiocam 506 color) and software (Zeiss Zen 2 blue edition).

### 4.6. Osteoblast Mineralization

SaOs-2 cells and human osteoblasts (PromoCell, cat. C-12720) were cultured to confluence in osteoblast growth medium (PromoCell, cat. C-27010) at 37 °C, 95% humidity and 5% CO_2_. Mineralization, deposit of hydroxyapatite, was investigated at cell confluency using mineralization medium (PromoCell, cat. nr. C27020) supplemented with recombinant cytokines; bone morphogenetic protein-2 (BMP-2) 5 ng/mL, TNF-α, IL-22 and IL-17A all at 1 ng/mL or a combination of the cytokines. The cells were cultured for 16 days with new medium, and cytokines added every 3 days. Cell mineralization was evaluated using the OsteoImage Bone Mineralization Assay (Lonza, Cat. PA-1503) according to the manufacturer’s guidelines and fluorescence was quantified using a Fluoroskan Ascent Microplate Fluorescence reader.

### 4.7. Proteomics

Serum samples were analyzed using Olink MOUSE EXPLORATORY assay (Proseek Multiplex, Olink Proteomics). BioXpedia analyzed 92 murine proteins, and each of the proteins are available at http://www.olink.com/products (28 June 2018). In brief, 1 μL sample was used for proximity extension immunoassay based on antibodies linked to DNA oligos analysis. Olink NPX manager software was used for processing of data. Data are presented as log2-transformed arbitrary units normalized protein expression (NPX). Public-access bioinformatic databases including Uniprot, Gene Ontology (GO), and STRING version 11.0 were used for the understanding of the biological relevance of selected differentially expressed proteins.

### 4.8. ELISA

ELISA was applied for additional analysis of RANKL expression. Serum RANKL levels were measured using (ab100749 RANKL Mouse Elisa kit, Abcam). The optical density was measured using Victor^3^ Multilabel plate counter (Perkin Elmer) at 450 nm, and the result was calculated using standard curve on a log–log scale.

### 4.9. Statistical Analysis

The statistical analysis was performed using Stata Statistical Software: release 13.1 (StataCorp LP). Parameters obtained from μCT data were not normally distributed and were statistically evaluated using Wilcoxon rank sum test for equal means on total bone volume between the groups at each time point. Data are presented as median ± SEM. ELISA s-RANKL data were analyzed using the Student’s *t*-test. For comparison of proteomic data, normality was checked using Shapiro–Wilks methods, histogram and QQ plots. Statistical analysis on normally distributed Olink Proseek data was performed using one-way ANOVA, and data not following normal distribution were compared using the Kruskal–Wallis rank test. Data are presented as mean ± SD; *p*-values < 0.05 were considered statistically significant.

## Data Availability

The authors declare that all relevant data supporting the findings of this study are available within the article and all data are available from the corresponding author on request.

## Supplementary Materials

The following are available online at https://www.mdpi.com/article/10.3390/ijms23010323/s1.
