# Supplementary material for: Intermittent Hypoxic Therapy Inhibits Allogenic Bone-Graft Resorption by Inhibition of Osteoclastogenesis in a Mouse Model
Source: Int J Mol Sci. 2021 Dec 28;23(1):323. doi: 10.3390/ijms23010323 (PMC8745522; doi:10.3390/ijms23010323)
Supplement: Supplementary file 1 [file ijms-23-00323-s001.zip › ijms-1483713-supplementary.pdf]

Supplementary Materials

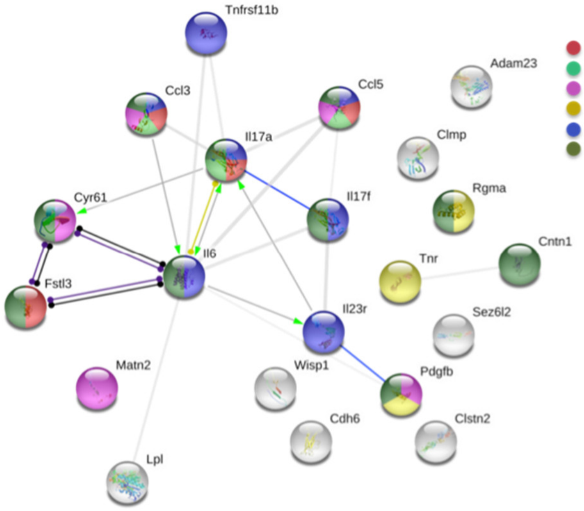

	Biological Pathway description - Control and Hypoxia	GO-term	Interating partners
(red)	Regulation of Osteoclast differentiation	GO:0045670	4
(green)	Positive regulation of Osteoclast differention	GO:0045672	3
(magenta)	Chemotaxis	GO:0006935	5
(yellow)	Negative regulation of response to wounding	GO:1903035	3
(blue)	Inflammatory response	GO:0006954	7
(dark green)	Postive regulation of cellular metabolic processes	GO:0045779	10

Figure S1. Key proteins of interest after 1 week. Graphical representation of biological pathways and interactions of significant proteins, control vs. IHT, after 1 week. The same coloring indicates the same biological pathway description. Data are extracted from STRING version 11.0 (string-db.org).

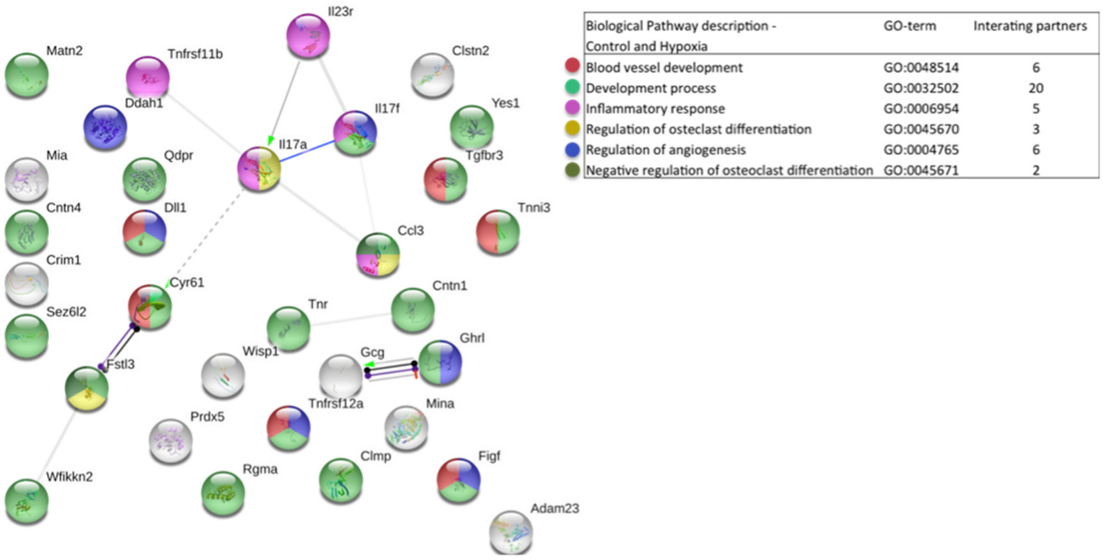

	Biological Pathway description - Control and Hypoxia	GO-term	Interating partners
(red)	Blood vessel development	GO:0048514	6
(green)	Development process	GO:0032502	20
(magenta)	Inflammatory response	GO:0006954	5
(yellow)	Regulation of osteclast differentiation	GO:0045670	3
(blue)	Regulation of angiogenesis	GO:0004765	6
(dark green)	Negative regulation of osteoclast differentiation	GO:0045671	2

Figure S2. Key proteins of interest after 4 weeks. Biological pathways and interactions of significant proteins, control vs. IHT, after 4 weeks. The same coloring indicates the same biological pathway description. Data are extracted from STRING version 11.0 (string-db.org).
